# How Can People Express Their Trait Self-Esteem Through Their Faces in 3D Space?

**DOI:** 10.3389/fpsyg.2021.591682

**Published:** 2021-02-04

**Authors:** Xiaoyang Wang, Xiaoqian Liu, Yuqian Wang, Tingshao Zhu

**Affiliations:** ^1^Institute of Psychology, Chinese Academy of Sciences, Beijing, China; ^2^Department of Psychology, University of Chinese Academy of Sciences, Beijing, China

**Keywords:** facial expressions, three-dimensional data, gender, logistic regression, trait self-esteem, two-way ANOVA

## Abstract

**Background:**

Trait self-esteem reflects stable self-evaluation, and it affects social interaction patterns. However, whether and how trait self-esteem can be expressed through behaviors are controversial. Considering that facial expressions can effectively convey information related to personal traits, the present study investigated the three-dimensional (3D) facial movements related to self-esteem level and the sex differences therein.

**Methods:**

The sample comprised 238 participants (46.2% males, 53.8% females). Their levels of trait self-esteem were evaluated by employing the Rosenberg Self-Esteem Scale (SES) (47.9% low self-esteem, 52.1% high self-esteem). During self-introductions, their facial movements in 3D space were recorded by Microsoft Kinect. Two-way ANOVA was performed to analyze the effect of self-esteem and gender on 3D facial movements. Additionally, Logistic regression models were established to describe the relationship between 3D facial movements and self-esteem levels in both genders.

**Results:**

The results of two-way ANOVA revealed a main effect of trait self-esteem level for cheeks and lips’ movements. Meanwhile, there was a significant interaction between trait self-esteem and gender on the variability of lips’ movements. In addition, the combination of facial movements can effectively identify trait self-esteem in men and women, with 75.5 and 68% accuracy, respectively.

**Conclusion:**

The present results suggest that the 3D facial expressions of individuals with different trait self-esteem levels were different, and such difference is affected by gender. Our study explores a possible way in which trait self-esteem plays a role in social interaction and also provides the basis for automatic self-esteem recognition.

## Introduction

Self-esteem is the positive or negative self-evaluation a person feels about oneself (Rosenberg, 1965), which can be divided into trait self-esteem (long-time fluctuations) and state self-esteem (short-time fluctuations) (Kernis, 1993). While state self-esteem is influenced by social evaluation (Leary et al., 2003), trait self-esteem is influenced by social evaluation and in turn affects social behavior (Greenwald and Banaji, 1995), social decision-making (Anthony et al., 2007), and ultimately, individual well-being (Baumeister et al., 2003). Individuals with higher trait self-esteem usually appear self-confident to others (Krause et al., 2016). They give people a more positive first impression (Back et al., 2011), are more likely to be admitted during interviews (Imada and Hakel, 1977), and have higher sales performance at work (Miner, 1962). Vice versa, perceptions of other’s self-esteem is an important factor in determining the mode of communication (Macgregor et al., 2013). One way to obtain an understanding of how trait self-esteem affects social interaction is to investigate the expression of trait self-esteem.

Previous studies have reported that males have slightly higher self-esteem than females (Feingold, 1994). Some comparisons showed that gender is an important factor in the relationship between self-esteem and body image (Israel and Ivanova, 2002), alcohol using (Neumann et al., 2009), and life satisfaction (Moksnes and Espnes, 2013). Besides, the self-reported personality traits of men and women with high self-esteem have weak differences (Robins et al., 2001). Agentic traits such as extraversion are important for men’s self-esteem, while communal traits such as agreeableness are important for women’s self-esteem. However, only a few studies have investigated sex differences in the expression of trait self-esteem. Considering that men and women with high self-esteem tend to show different traits and do different behaviors, they are much likely to show their trait self-esteem in different ways.

Many researchers have studied the perception of self-esteem instead of the expression of self-esteem, yet different results have been obtained. A study revealed that self-esteem may not be perceived well through explicit behavioral clues (e.g., maintain eye contact and hesitate when speaking) (Savin-Williams and Jaquish, 1981). Other studies indicated zero correlation between self-reported self-esteem and others’ perceived self-esteem (Yeagley et al., 2007; Kilianski, 2008). However, a recent study has shown that when controlling acquaintance, people can identify trait self-esteem from self-introduction videos fairly accurately (Hirschmueller et al., 2018). The perception of self-esteem is influenced by prior knowledge and individual differences (Ostrowski, 2010; Nicole et al., 2015), and it is difficult to identify fine-grained behavioral information. Using advanced technology to encode the expression of trait self-esteem may be an objective way to study the interpersonal effects of trait self-esteem. For example, the relationship between encoded gait information and trait self-esteem was found in previous research (Sun et al., 2017).

Compared with other expressions, facial movements convey more information about personal traits (Harrigan et al., 2004) and are more noticed. Meanwhile, facial expressions play an important role in social interaction and communication (Berry and Landry, 1997; Ishii et al., 2018). As a result, facial movement is likely to be an aspect of trait self-esteem expression. On one hand, facial movements are related to personality and depression (de Melo et al., 2019; Suen et al., 2019), which are linked to self-esteem (Robins et al., 2001). On the other hand, gender differences exist in facial movements (Hu et al., 2010). Taken together, these previous findings provide evidence for the sex differences in the facial movements related to trait self-esteem. Therefore, it is important to distinguish the unique facial expressions of men and women with high self-esteem/low self-esteem. Moreover, three-dimensional (3D) facial data are generally considered to contain more information than 2D facial data and have better performance in identifying traits (Jones et al., 2012; de Melo et al., 2019). Thus, it is more comprehensive to explore the facial expressions of trait self-esteem in 3D space.

The purpose of this study was to investigate the 3D facial movements related to trait self-esteem and whether such relationship is influenced by gender. Based on previous findings, we hypothesized that facial movements would be separately influenced by trait self-esteem level and gender. In addition, we expected interaction exists between self-esteem level and gender in their effect on facial movements.

## Materials and Methods

### Participants

A total of 240 students and workers from University of Chinese Academy of Sciences participated in this study. To participate in the study, participants had to meet three inclusion criteria: (1) At least 18 years old; (2) Fluent in Mandarin; and (3) Healthy and able to make normal facial expressions. After excluding the data of participants who frequently covered their faces with their hands, 238 qualified samples were obtained, of which 110 were male and 128 were female. The retention rate was 99.17%. The average age was 22.8 years (SD = 2.8).

A post hoc G power analysis (Faul et al., 2007) on 238 participants was conducted with *a priori* alpha levels of 0.05 and a medium effect. This power analysis demonstrated relatively large effects (effects of power = 0.97) (MacCallum et al., 1996).

### Measures

#### Rosenberg Self-Esteem Scale

This study used the Chinese version of the Rosenberg Self-Esteem Scale (SES) (Rosenberg, 1965; Ji and Yu, 1993) as a tool to measure trait self-esteem (Kang, 2019). The scale contains 10 questions in total and the score range from 10 to 40; the higher the score, the higher the level of self-esteem. Generally speaking, scores lower than 15 indicate very low self-esteem, scores range in 15–20 indicate low self-esteem, scores range in 20–30 indicate normal self-esteem, scores range in 30–35 indicate high self-esteem, and scores above 35 indicate very high self-esteem. In this study, we divided the subjects into two groups based on their self-esteem score. Scores higher than 30 were classified into high self-esteem group (HSE), and scores lower than or equal to 30 were classified into low self-esteem group (LSE).

#### Kinect

**FIGURE 1:**
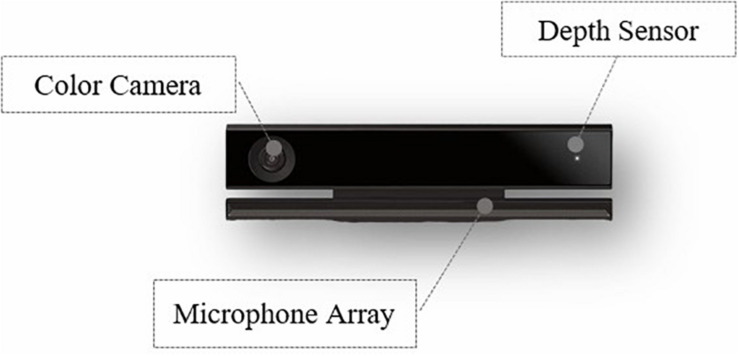
The Kinect camera and a brief introduction about its main components.

**FIGURE 2:**
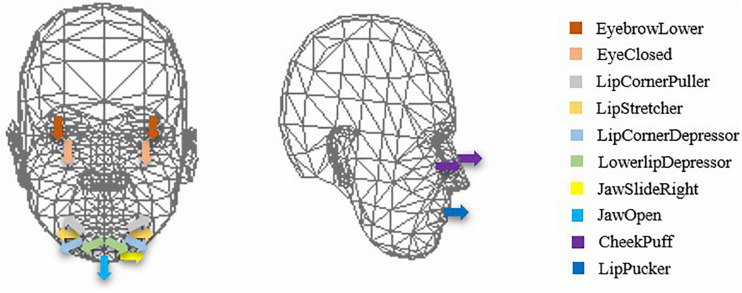
The schematic diagram of each AU.

A Kinect camera can be used to recognize facial movements automatically (Zaletelj and Koir, 2017) (Figure 1). The sampling frequency of the Kinect is 30 Hz, which means it can record 30 frames of individual facial activity per second. To quantitatively describe the 3D facial movements, Kinect is able to capture and track the key 17 action units (AUs) (Ekman and Friesen, 1978) around facial features in 3D space. The specific meaning of each AU is provided in Table 1, and the intuitive facial movements are shown in Figure 2, which shows that there are three AUs moving in depth dimension. Most of the AUs are expressed as a numeric weight that varies between 0 and 1. Three of the AUs – jaw slide right, right eyebrow lower, and left eyebrow lower – vary between −1 and +1. The absolute value of the numeric weight represents the moving distance of the corresponding AU. Finally, Kinect will collect one list of time series data for each AU’s movements.

**TABLE 1:** The specific meaning of each AU.

Facial action units	Action description	Value range
JawOpen	Dropping of the chin without opening the mouth	(0, 1)
LipPucker	Pouting	(0, 1)
JawSlideRight	Slide the chin to the right (the negative number indicates a sliding of the chin to the right and then to the left)	(−1, 1)
LipStretcherRight	Stretch the right corners of mouth to the right	(0, 1)
LipStretcherLeft	Stretch the left corners of mouth to the left	(0, 1)
LipCornerPullerLeft	Pull the left corners of mouth to the upper left	(0, 1)
LipCornerPullerRight	Pull the right corners of mouth to the upper right	(0, 1)
LipCornerDepressorLeft	Pull the left corners of mouth to the left and down	(0, 1)
LipCornerDepressorRight	Pull the right corners of mouth to the right and down	(0, 1)
LeftcheekPuff	Puff up the left cheek	(0, 1)
RightcheekPuff	Puff up the right cheek	(0, 1)
LefteyeClosed	Close the left eye	(0, 1)
RighteyeClosed	Close the right eye	(0, 1)
RighteyebrowLower	Right eyebrow down (the negative number indicates a raised right eyebrow)	(−1, 1)
LefteyebrowLower	Left eyebrow down (the negative number means the left eyebrow is raised)	(0, 1)
LowerlipDepressorLeft	Pull the lower lip down to the left	(0, 1)
LowerlipDepressorRight	Pull the lower lip down to the right	(0, 1)

*The value represents the size of the facial action units’ movements in proportion to the resting state, usually 0.8 means a very large movement.*

### Design and Procedure

To analyze the relationship between self-esteem and 3D facial movements, this study designed a facial data collection experiment with a self-introductory situation according to the following steps: (1) First, the demographic information of a subject was recorded. (2) Then, the level of self-esteem was determined using the SES. (3) During the experimental task (self-introduction), the participants’ facial changes in 3D space (the movement of AUs) were captured by Kinect.

Specifically, during the experimental task, a self-introductory situation was set. Compared with conversation contexts and emotional-induction scenes, self-introduction facing the camera is least affected by social evaluation. We believe that in this situation, the subjects show their trait self-esteem rather than state self-esteem (Park et al., 2007). In this section, participants were asked to make a 1-min self-introduction using the following outline: (1) Please introduce yourself and your hometown in detail; (2) Please give a detailed account of your major and the interesting research work you did when you were at school; (3) Please describe your plans for the future and what kind of work you would like to do. Meanwhile, illumination, the relative position, and the relative distance between Kinect and participants were strictly controlled. For illumination, we chose a bright room without direct sunlight for data acquisition; for relative position, we asked the participants to keep their body as still as possible; for distance, we controlled the distance between the camera and the participants to 3 m.

**FIGURE 3:**
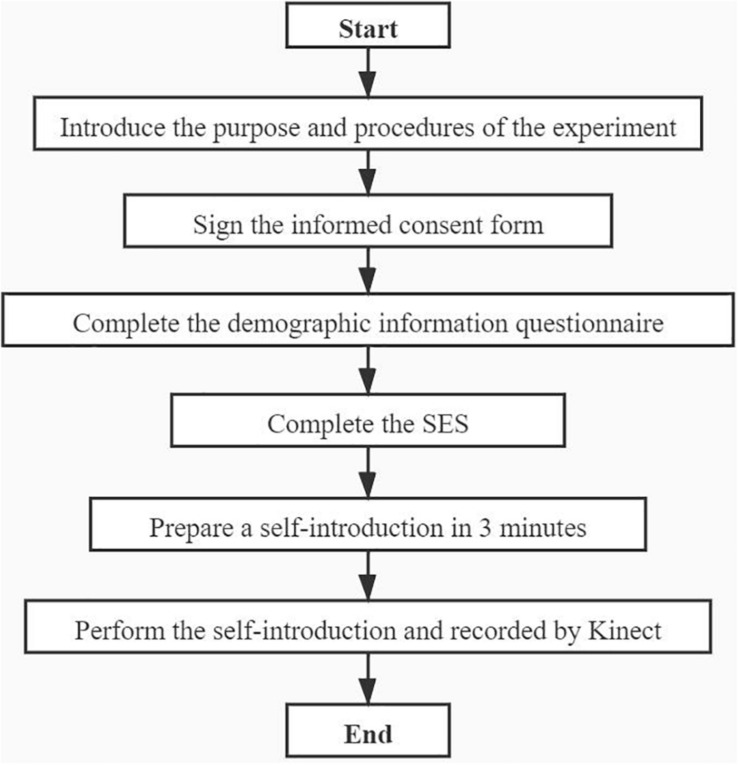
The whole process of the experiment.

The whole process of the experiment is shown in Figure 3. The same steps were followed for each subject independently. The entire process took approximately 10 min.

### Statistical Analysis

After the experiment, we collected demographic information, self-esteem score, and 3D facial movements for each subject. Then we performed data preprocessing. For self-esteem scores, people in the LSE group were defined as those whose self-esteem score was less than or equal to 30 points, while the HSE group had a score greater than 30. For AU data, we normalized the AU data and eliminated outliers outside of the three standard deviations. Then the average and standard deviation values of each AU were calculated to describe the magnitude and variability of the facial muscle’s movements. The greater the absolute value, the larger the moving amplitude. In addition, the greater the standard deviation, the larger the facial variability.

For each AU indicator, descriptive analyses were used to calculate the means and SDs for each group (LSE vs HSE), and the data were also analyzed according to gender (male vs female). Considering the ratio of the number of variables to the sample size, differences between the mean scores for group × gender were calculated using the two-way ANOVA. Then, multivariate analyses using logistic regression models were conducted to identify the relationship between AUs’ movements level and self-esteem level in which odds ratios (ORs) were calculated. In addition, stepwise logistic regressions were followed for removing unimportant independent variables to get the best-fitted models. All statistical processes were performed on SPSS 22.0, with a two-tailed probability value of <0.05 considered to be statistically significant.

## Results

A total of 110 males (46.22%) and 128 (53.78%) were involved in this study. No significant sex × SE-level differences in age distribution were found. The mean values as well as standard deviation values of AU movements are described as M ± SD in Table 2. The significant results from a 2 × 2 ANOVA separated by SE level (i.e., LSE and HSE) and sex (i.e., male and female) are shown in Table 3. Significant models to predict self-esteem level from AU movements are shown in Table 4.

**TABLE 2:** Participants characteristics.

	Male (*n* = 110)		Female (*n* = 128)	
	LSE (*n* = 50)	HSE (*n* = 60)	LSE (*n* = 64)	HSE (*n* = 64)
	M ± SD	M ± SD	M ± SD	M ± SD
Age	22.84 ± 1.973	23.33 ± 3.079	21.98 ± 2.504	23.17 ± 3.215
SES score	27.00 ± 3.369	34.77 ± 2.513	26.66 ± 3.433	34.31 ± 2.085
**Mean value of facial AUs**				
Jaw_open_mean	0.105 ± 0.056	0.113 ± 0.061	0.102 ± 0.062	0.104 ± 0.055
LipPucker_mean	0.383 ± 0.217	0.425 ± 0.195	0.409 ± 0.214	0.393 ± 0.184
JawSlideRight_mean	0.012 ± 0.07	-0.001 ± 0.063	0.013 ± 0.055	0.006 ± 0.058
LipStretcherRight_mean	0.094 ± 0.081	0.123 ± 0.1	0.083 ± 0.087	0.09 ± 0.08
LipStretcheLeft_mean	0.102 ± 0.092	0.125 ± 0.093	0.11 ± 0.092	0.121 ± 0.087
LipCornerPullerLeft_mean	0.128 ± 0.129	0.133 ± 0.123	0.156 ± 0.134	0.127 ± 0.131
LipCornerPullerRight_mean	0.147 ± 0.123	0.168 ± 0.13	0.197 ± 0.154	0.162 ± 0.138
LipCornerDepressorLeft_mean	0.131 ± 0.086	0.101 ± 0.072	0.136 ± 0.085	0.12 ± 0.087
LipCornerDepressorRight_mean	0.126 ± 0.084	0.111 ± 0.095	0.146 ± 0.101	0.131 ± 0.096
LeftcheekPuff_mean	0.19 ± 0.171	0.278 ± 0.177	0.2 ± 0.193	0.228 ± 0.18
RightcheekPuff_mean	0.228 ± 0.194	0.308 ± 0.195	0.216 ± 0.208	0.255 ± 0.226
LefteyeClosed_mean	0.264 ± 0.13	0.289 ± 0.142	0.267 ± 0.136	0.253 ± 0.113
RighteyeClosed_mean	0.356 ± 0.158	0.36 ± 0.158	0.348 ± 0.168	0.319 ± 0.128
RighteyebrowLower_mean	0.181 ± 0.311	0.233 ± 0.371	0.2 ± 0.289	0.105 ± 0.292
LefteyebrowLower_mean	0.197 ± 0.343	0.269 ± 0.391	0.243 ± 0.322	0.138 ± 0.333
LowerlipDepressorLeft _mean	0.038 ± 0.049	0.039 ± 0.039	0.071 ± 0.074	0.048 ± 0.046
LowerlipDepressorRight_mean	0.047 ± 0.054	0.044 ± 0.035	0.074 ± 0.066	0.056 ± 0.05
**Std value of facial AUs**				
Jaw_open_std	0.045 ± 0.015	0.048 ± 0.015	0.055 ± 0.016	0.055 ± 0.016
LipPucker_std	0.094 ± 0.039	0.111 ± 0.049	0.127 ± 0.052	0.125 ± 0.044
JawSlideRight_std	0.049 ± 0.02	0.054 ± 0.022	0.05 ± 0.017	0.046 ± 0.013
LipStretcherRight_std	0.056 ± 0.034	0.068 ± 0.038	0.055 ± 0.037	0.062 ± 0.039
LipStretcheLeft_std	0.056 ± 0.031	0.067 ± 0.033	0.062 ± 0.035	0.066 ± 0.033
LipCornerPullerLeft_std	0.086 ± 0.056	0.094 ± 0.058	0.116 ± 0.069	0.09 ± 0.07
LipCornerPullerRight_std	0.089 ± 0.054	0.103 ± 0.057	0.117 ± 0.064	0.096 ± 0.06
LipCornerDepressorLeft_std	0.077 ± 0.033	0.072 ± 0.046	0.087 ± 0.038	0.073 ± 0.037
LipCornerDepressorRight_std	0.075 ± 0.032	0.074 ± 0.045	0.086 ± 0.038	0.077 ± 0.04
LeftcheekPuff_std	0.062 ± 0.049	0.089 ± 0.045	0.076 ± 0.05	0.081 ± 0.041
RightcheekPuff_std	0.091 ± 0.065	0.112 ± 0.051	0.091 ± 0.054	0.093 ± 0.057
LefteyeClosed_std	0.098 ± 0.03	0.108 ± 0.028	0.112 ± 0.029	0.116 ± 0.028
RighteyeClosed_std	0.115 ± 0.036	0.119 ± 0.031	0.123 ± 0.033	0.13 ± 0.034
RighteyebrowLower_std	0.084 ± 0.037	0.089 ± 0.045	0.095 ± 0.045	0.087 ± 0.034
LefteyebrowLower_std	0.090 ± 0.047	0.092 ± 0.047	0.097 ± 0.044	0.096 ± 0.041
LowerlipDepressorLeft_std	0.039 ± 0.044	0.048 ± 0.041	0.073 ± 0.056	0.053 ± 0.045
LowerlipDepressorRight_std	0.041 ± 0.048	0.046 ± 0.037	0.070 ± 0.052	0.053 ± 0.045

*LSE, low trait self-esteem group; HSE, high trait self-esteem group; _mean, the mean value of the AU’s corresponding temporal records; _std, the standard deviation of the AU’s corresponding temporal records.*

**TABLE 3:** Significant results for two-way analysis of variance.

Variance	Variable	SS	*F*	*P*	η^2^	Pairwise comparisons
Sex	LowerlipDepressorLeft _mean	0.026	8.716**	0.003	0.036	Males < females
	LowerlipDepressorRight_mean	0.023	8.485**	0.004	0.035	Males < females
	Jaw_open_std	0.004	16.951***	0.000	0.068	Males < females
	LipPucker_std	0.033	15.169***	0.000	0.061	Males < females
	LefteyeClosed_std	0.008	9.426**	0.002	0.039	Males < females
	RighteyeClosed_std	0.006	5.312*	0.022	0.022	Males < females
	LowerlipDepressorLeft_std	0.022	9.956**	0.002	0.041	Males < females
	LowerlipDepressorRight_std	0.018	8.668**	0.004	0.036	Males < females
SES level	LipCornerDepressorLeft_mean	0.030	4.386*	0.037	0.018	LSE > HSE
	LeftcheekPuff_mean	0.199	6.080*	0.014	0.025	LSE < HSE
	RightcheekPuff_mean	0.212	4.945*	0.027	0.021	LSE < HSE
	LipStretcherRight_std	0.005	3.865	0.050	0.016	LSE < HSE
	LeftcheekPuff_std	0.014	6.801*	0.010	0.028	LSE < HSE
Sex × SES level	LipCornerPullerLeft_std	0.016	3.961*	0.048	0.017	LSE:Male < HSE:Male LSE:Female > HSE:Female
	LipCornerPullerRight_std	0.017	4.975*	0.027	0.021	LSE:Male < HSE:Male LSE:Female > HSE:Female
	LowerlipDepressorLeft_std	0.013	5.870*	0.016	0.024	LSE:Male < HSE:Male LSE:Female > HSE:Female

SS, sum of squares; LSE, low trait self-esteem group; HSE, high trait self-esteem group. *P < 0.05; **P < 0.01; ***P < 0.001.

**TABLE 4:** The relationship between AUs’ movement and self-esteem.

Gender	Variates	β	95% CI	*P* value
Male	LipPucker_mean			0.022*
	LipPucker_mean_1_	0.347	0.093–1.293	0.115
	LipPucker_mean_2_	3.079	0.695–13.645	0.139
	LipPucker_mean_3_	0.765	0.210–2.793	0.686
	LeftcheekPuff_mean			0.003**
	LeftcheekPuff_mean_1_	0.075	0.018–0.310	0.000***
	LeftcheekPuff_mean_2_	0.483	0.124–1.871	0.292
	LeftcheekPuff_mean_3_	0.248	0.070–0.877	0.031*
	LipCornerDepressorLeft_std			0.008**
	LipCornerDepressorLeft_std_1_	2.482	0.565–10.915	0.229
	LipCornerDepressorLeft_std_2_	0.249	0.060–1.031	0.055
	LipCornerDepressorLeft_std_3_	0.403	0.101–1.612	0.199
Female	LefteyebrowLower_mean			0.016**
	LefteyebrowLower_mean_1_	5.553	0.823–5.866	0.116
	LefteyebrowLower_mean_2_	2.389	1.849–15.123	0.002**
	LefteyebrowLower_mean_3_	1.383	0.549–3.939	0.443
	LipCornerDepressorLeft_std			0.013*
	LipCornerDepressorLeft_std_1_	3.478	0.559–9.882	0.037*
	LipCornerDepressorLeft_std_2_	5.507	0.116–2.096	0.003**
	LipCornerDepressorLeft_std_3_	1.261	0.112–1.902	0.663

AU_mean(std)_1_, Q0–Q25 score section; AU_mean(std)_2_, Q25–Q50 score section; AU_mean(std)_3_, Q50–Q75 score section. *P < 0.05; **P < 0.01; ***P < 0.001.

There were significant main effects for the SE levels: LipCornerDepressorLeft_mean [*F* (1, 234) = 4.386, *P* = 0.037], LeftcheekPuff_mean [*F* (1, 234) = 6.080, *P* = 0.014], RightcheekPuff_mean [*F* (1, 234) = 4.945, *P* = 0.027], LipStretcherRight_std [*F* (1, 234) = 3.865, *P* = 0.050], and LeftcheekPuff_std [*F* (1, 234) = 6.801, *P* = 0.010]. The results revealed that amplitude and richness of facial movements increased as self-esteem became higher, except the amplitude of LipCornerDepressorLeft_mean AU.

There were also significant interaction effects between sex and self-esteem level: LipCornerPullerLeft_std [*F* (1, 234) = 3.961, *P* = 0.048], LipCornerPullerRight_std [*F* (1, 234) = 4.975, *P* = 0.027], and LowerlipDepressorLeft_std [*F* (1, 234) = 5.870, *P* = 0.016]. These three AUs in men with low self-esteem moved more variably than men with high self-esteem, while in women, the opposite was true.

Step-forward logistic regression models were conducted to check whether some crucial AUs’ movements could combine to predict self-esteem as the optimal model in both genders. Table 4 presented the multivariable associations between AUs’ movements in 3D space and self-esteem. Three independent variables were included in the logistic regression model of men, they were LipPucker_mean (*P* = 0.022), LeftcheekPuff_mean (*P* = 0.003), and LipCornerDepressorLeft_std (*P* = 0.008). In addition, two independent variables were included in the logistic regression model of women, they were LefteyebrowLower_mean (*P* = 0.016) and LipCornerDepressorLeft_std (*P* = 0.013). Those AUs’ indicators might be powerful when they are combined to predict self-esteem. Both logistic regression models were significant (*P* < 0.001), with men and women models having prediction accuracy in classification of 75.5 and 68%, respectively. In addition, we made a further confirmation that the effect of building models separately for men and women was better than building a combining model (shown in Table 5).

**TABLE 5:** The results of logistic regression models.

	Accuracy^*a*^	Precision^*b*^	Recall^*c*^
Model for all	67.2	65.0	68.4
Model for female	68.0	67.1	70.3
Model for male	75.5	76.7	66.0

*^*a*^The number of correctly classified/the total number of samples. ^*b*^The number of LSE samples correctly classified/the total number of samples classified as LSE. ^*c*^The number of LSE samples correctly classified as positive/the total number of LSE samples.*

## Discussion

The purpose of the study was to explore the relationship between 3D facial movements and trait self-esteem. Kinect was employed to quantify facial movements during self-introduction and statistical analysis explored the relationship between facial movements and level of self-esteem. The results were concordant with our hypothesis, revealing that some 3D facial movements were related to trait self-esteem, and sex factor has an impact on such relationship.

Previous studies found that self-esteem may not be perceived well through explicit behavioral clues (Savin-Williams and Jaquish, 1981; Kilianski, 2008). In contrast, the present findings indicate that during self-introductions, the trait self-esteem has a great effect on some facial AUs’ movements, and a specific combination of facial AUs’ movements can effectively predict the level of trait self-esteem. This extends a recent work that self-esteem perceptions of strangers based on self-introduction videos were fairly accurate (*r* = 0.31, *P* = 0.002) (Hirschmueller et al., 2018). Specifically, compared with the LSE group, individuals in the HSE group puff their cheeks larger, move their lips to right more, and puff their left cheek more. Differently, high self-esteem people move their lip corner to bottom left smaller, which is a very unique movement best representing sadness (coded as AU15 in FACS) (Ekman and Friesen, 1978). One potential explanation is that trait self-esteem may be expressed through different emotions during self-rating. While individuals with high self-esteem tend to express more positive emotions such as pride (Tracy and Robins, 2003; Agroskin et al., 2014) and happiness (Kim et al., 2019) when introducing themselves, those with low self-esteem are more likely to exhibit negative emotions including sadness and disgust (Satoh, 2001). In addition, it is noteworthy that the left cheek was found to be the area most affected by trait self-esteem (for amplitude: η^2^ = 0.025, for variability: η^2^ = 0.028). This led to the hypothesis that trait self-esteem may be a potential factor for the left cheek bias (Nicholls et al., 2002; Lindell and Savill, 2010; Lindell, 2013). Future factor analysis researches on left cheek bias can take trait self-esteem into consideration.

Moreover, the interaction effect exists in the influence of trait self-esteem and gender on facial movements. In terms of the variability of lip movements, high self-esteem males move more variable than low self-esteem males, while low self-esteem females move more variable than high self-esteem females. One way to obtain an understanding of why men and women express their trait self-esteem differently is that self-esteem is largely derived from interpersonal experience (Leary et al., 1995) and gender identity (Cvencek et al., 2016). Therefore, men who are more deterrent enjoy higher social status and thus, higher levels of self-esteem. On the contrary, women who have more affinity have enhanced interpersonal relationships and thus, higher levels of self-esteem (Baldwin and Keelan, 1999). An alternative explanation is that men and women with high self-esteem have different personality traits. While agentic traits such as extraversion are more important for men’s self-esteem, communal traits (e.g., agreeableness) are more crucial for women’s self-esteem (Robins et al., 2001). Although the mechanism is unclear, our study indicates that the facial expressions of trait self-esteem are related to gender, and establishing self-esteem prediction models according to gender is better than combined prediction. It is recommended that future studies on self-esteem should consider gender as an important factor.

The following briefly foregrounds some of the study’s implications. First, the present study collected reasonable self-introduction video data and employed advanced 3D facial movement quantification techniques to determine the relationship between 3D facial expressions and trait self-esteem. To the authors’ knowledge, it is the first to investigate the quantified 3D facial expressions of trait self-esteem, and our results demonstrate the necessity. Second, our research discovers the effect of gender on the facial expression of self-esteem and provided suggestions for future nonverbal research on self-esteem. Finally, the results provide a basis for predicting self-esteem based on 3D facial data. In view of the possibility that the self-reporting method may result in false answers during interviews and job scenarios (McLarnon et al., 2019), a facial prediction method offers a supplementary strategy to measure self-esteem.

However, the findings must be considered in light of various limitations. First of all, since this study was conducted in China, how many findings can be extended to other cultures is uncertain. Previous studies have indicated that Asian subjects may have different facial expressions than subjects from other cultural backgrounds (Matsumoto and Ekman, 1989). In addition, cultures differed significantly in the magnitude of gender effect on self-esteem. Future cross-cultural research might help to understand the difference in facial expression of self-esteem between cultures. Secondly, in accordance with theories of the lens model (Watson and Brunswik, 1958), the influence of trait self-esteem in social interaction is realized through the expression of trait self-esteem and the perception of such expression. This study only objectively explored the relationship between facial expressions and self-esteem but did not investigate the extent to which human perceivers can be affected by these expressions. A further limitation can be seen in the influence of participants’ knowledge background in self-reporting methods.

Regarding the future lines of research, stable trait self-esteem prediction models can be proposed based on researches about trait self-esteem expression. Considering social approval and knowledge background, automatic self-esteem recognition can be used to measure self-esteem in interview scenarios or special populations. Furthermore, multimodal data (such as eye movements, facial movements, and gestures) can be combined to fully describe the expression of self-esteem and establish more accurate prediction models.

## Data Availability Statement

The datasets presented in this article are not readily available because raw data cannot be made public. If necessary, we can provide behavioral characteristic data. Requests to access the datasets should be directed to XL.

## Ethics Statement

The studies involving human participants were reviewed and approved by the scientific research ethics committee of the Chinese Academy of Sciences Institute of Psychology (H15010). The patients/participants provided their written informed consent to participate in this study.

## Author Contributions

TZ contributed to the conception and design of the study. XL collected the data and developed the instrument. XW performed most of the statistical analysis and wrote the manuscript with input from all authors. YW performed part of the statistical analysis and helped drafting a part of the text. All authors contributed to the article and approved the submitted version.

## Conflict of Interest

The authors declare that the research was conducted in the absence of any commercial or financial relationships that could be construed as a potential conflict of interest.
